# Prenatal Testosterone Exposure Worsen the Reproductive Performance of Male Rat at Adulthood

**DOI:** 10.1371/journal.pone.0071705

**Published:** 2013-08-15

**Authors:** Fahimeh Ramezani Tehrani, Mahsa Noroozzadeh, Saleh Zahediasl, Asghar Ghasemi, Abbas Piryaei, Fereidoun Azizi

**Affiliations:** 1 Reproduction Endocrinology Research Center, Research Institute for Endocrine Sciences, Shahid Beheshti University of Medical Sciences, Tehran, Iran; 2 Endocrine Physiology Research Center, Research Institute for Endocrine Sciences, Shahid Beheshti University of Medical Sciences, Tehran, Iran; 3 Department of Biology and Anatomical Sciences, Faculty of Medicine, Shahid Beheshti University of Medical Sciences, Tehran, Iran; 4 Endocrine Research Center, Research Institute for Endocrine Sciences, Shahid Beheshti University of Medical Sciences, Tehran, Iran; University of Southampton, United Kingdom

## Abstract

The reproductive system is extremely susceptible to environmental insults, for example exogenous steroids during gestational development and differentiation. Experimental induction of androgen excess during prenatal life in female animal models reprograms their reproductive physiology, however the fetal programming of the male reproductive system by androgen excess has not been well studied. We aimed to determine the effect of prenatal exposure of two different doses of testosterone on different gestational days, on the male reproductive system using a rat model. Sixteen pregnant rats were randomly divided into two experimental groups and two control groups. Experimental group І were subcutaneously injected with 3 mg free testosterone on gestational days 16-19 and its controls received solvent for that time; experimental group П were subcutaneously injected with 20 mg free testosterone on day 20 of gestational period and its controls received solvent at the same time. The reproductive system morphology and function of 32 male offspring of these study groups were compared at days 6-30-60 of age and after puberty. The anogenital distance of the male offspring of both experimental groups had no significant differences on the different days of measurement, compared with controls. In the offspring of experimental group І, the testes weight, number of Sertoli, Spermatocyte and Spermatid cells, sperm count and motility and the serum concentration of testosterone after puberty were significantly decreased; except for reduction of sperm motility (p< 0.01), the other effects were not observed in the offspring of experimental group ІІ. In summary, our data show that prenatal exposure of male rat fetuses to excess testosterone disrupted reproductive function, an effect highly dependent on the time, duration and level of exposure. It seems that the reproductive system in individuals exposed to high levels of androgens during fetal life should be evaluated at puberty and likely to be treated.

## Introduction

The reproductive system is extremely susceptible to environmental insults, for example exogenous steroids during gestational development and differentiation that this effect is highly dependent on the time, duration and level of exposure [1–3].

Exposure to exogenous steroids, due to industrial pollutants [4], could affect the developing fetus. Furthermore the fetuses of women with polycystic ovary syndrome (PCOS), the most common reproductive endocrinopathy, are exposed to elevated levels of androgens [5]. Although there are several studies that demonstrated the impact of prenatal exposure of female fetuses to excess androgen, such studies in male offspring are limited.

Experimental induction of androgen excess during prenatal or early postnatal life, in female animal models has been shown to reprogram reproductive and metabolic physiology, resulting in irregular, intermittent or absent estrous cycles, hyperandrogenism and polycystic ovarian morphology [3,6], manifestations are highly dependent on the time and the amount of androgens used. It has been demonstrated that androgen-mediated development of a normal male reproductive system occurs via androgen "programming" within a specific fetal time window (embryonic days 15.5-18.5) in the rat, that precedes morphological differentiation and development of the relevant tissues [7].

Androgens play a role not only in genital tract differentiation, phenotypic virilization and maintenance of secondary male characteristics, but also in the initiation and maintenance of spermatogenesis [8]. There is no agreement about the morphological and functional effects of increased androgen exposure in male animal models, but some studies report that increased androgen exposure during fetal male sexual differentiation altered testicular size, testosterone concentration, seminiferous tubule size and function, germ cell number, sperm count and motility [1,9–11]. The exact mechanisms of the above mentioned pathologies have not been well explained; the occlusion of the lumen of seminiferous tubules could be a possible mechanism reported by one study [1], however others explained that excess androgen could possibly decrease the amount of endogenous androgens through its negative feedback on gonadotropin secretion [12]. It has been demonstrated that endogenous androgens have an indirect important role in Sertoli cells proliferation via the androgen receptor-positive peritubular myoid cells, a possible mechanism that further explained the impact of prenatal androgen exposure [13,14]. Despite these explanations, the fetal programming of male reproductive system following androgen excess has not been well studied. It is not ethically approved to evaluate the effect of androgen excess during human pregnancy on adult reproduction function. Due to great similarities of genetic background of human and rats, they could considered as an appropriate animal model for exploring these aspects of fetal programming that has not well known. As a result of lack of proper evidence on the effect of prenatal exposure of male fetus to excess androgens and controversy findings observed in existed animal models, we aimed to determine the effect of prenatal exposure of two different doses of testosterone on different gestational days, on the male reproductive system (sperm quality, Sertoli cell number and hormone levels), using a rat model.

## Materials and Methods

### Animals

Sixteen adult female Wistar rats with 170-190 (g) body weight were supplied by the animal center of the Research Institute for Endocrine Sciences (RIES), Shahid Beheshti University of Medical Sciences (Tehran, Iran). Each female rat was housed in a separate polypropylene cage (43cm×30cm×15cm) with a male rat (1 female with 1 male) overnight in an environmentally controlled room (temperature 22± 3°C, relative humidity 24± 6% with 12-h light/dark cycles, light period beginning at 0700 a.m). Observation of the vaginal plug was considered as the first day of pregnancy. Sixteen pregnant rats were randomly divided into four groups (four pregnant rats in each group) that these groups were including; experimental groups І and П and vehicle groups or control groups І and П.

### Ethics statement

Animals were handled according to the principles of laboratory animal care approved by the local ethics committee of the RIES (Permit Number: 426 EC 91.06.07), Shahid Beheshti University of Medical Sciences (Tehran, Iran).

### Hormone dosage and injections

The doses of testosterone and their injection time were chosen according to previous studies; these doses of androgen induced reproductive disorders in female rats [3,15].

In experimental group І, pregnant animals were injected subcutaneously with 3 mg free testosterone (T1500, Sigma, Germany) dissolved in 500µl of sesame oil (S3547, Sigma, Germany) and benzyl benzoate (B6630, Sigma, Germany) with ratio 4:1, on gestational days 16-19, daily (n=4) and each pregnant animal in its vehicle group or control group І (n=4) was injected 500µl solvent (sesame oil and benzyl benzoate) simultaneously using the same method.

In experimental group П, pregnant animals were injected subcutaneously with 20 mg of free testosterone dissolved in 1 ml sesame oil and benzyl benzoate on day 20 of gestational period (n=4), while its vehicle group or control group П received 1ml solvent (n=4).

### Determination of the male offspring percent and its mortality rate

In control group І, total number of offspring were 42 (19 male + 23 female), in experimental group І, total number of offspring were 49 (22 male + 27 female), in control group П, total number of offspring were 39 (23 male + 16 female) and in experimental group П, total number of offspring were 30 (12 male + 18 female). The percentage of male offspring in each group was determined at the first day of birth, separately (Sex male distribution in the each group, number of male offspring in each group/total number offspring in each group), and mortality rate of male offspring were determined until days 30, 60 and 120 of age in each group, separately (total dead male number per testing day/total number of male offspring at the first day of birth).

### Animal selection for this study

Thirty-two male animals from four groups of study (control and fetal hyperandrogenemia), in other words eight male offspring in each group (two male offspring from each mother) were selected and were housed in the cages with free access for food and water, to this study. These thirty-two male animals were used before and after puberty (120-130 days of age) to evaluate of morphology and function of its reproductive system and other parameters.

### Measurement of anogenital distance and determination of body weight

Anogenital distance (AGD, is distance (mm) from cranial edge of anus to base of phallus) of male animals, in each group were determined by vernier calipers on days 6, 30, 60 after birth and after puberty; also, the body weight of male animals from all groups were recorded, immediately after birth, on days 15, 30, 45, 60 of age and after puberty.

### Preparation of sperm samples

Sperm solution preparation were carried out according to the method described by Giribau, N et al. [16] with some modification. In brief, animals were anesthetized by intraperitoneal injection (i.p) of pentobarbital sodium (P3761, 5mg, Sigma, USA) dissolved in normal saline 0.9% (60 mg/kg body weight), immediately after anesthesia; the right epididymis was removed, weighed and then transferred to petri dish containing 2 ml of normal saline, caudal portion of epididymis isolated, split with needle then 20 µl semen removed from caudal portion of epididymis. Semen was diluted in 4 ml normal saline at temperature of 34^°^ C.

### Sperm motility

Evaluation of the sperm motility, was carried out according to the method described by Giribau, N et al and Toledo, F.C et al [16,17] with some modification. In brief, immediately after preparation of sperm solution, one drop of sperm solution was placed on Neubauer Chamber slide and 10 fields were examined, under a light microscope (100 x magnification). Motility of sperm was classified as: Type A motile, with fast progressive trajectory; type B motile, with slow progressive trajectory; type C sluggish movement and type D immotile. The entire process of sperm motility was performed within 5 min after isolation from cauda epididymis. First, non motile sperms were counted followed by motile sperms. Sperm motility was expressed as a percentage of total sperm count.

### Sperm count

One drop from sperm solution was taken and transferred to a Neubauer Chamber slide (four fields of W.B.C) and the number of sperms were counted using a light microscope (100 x magnification) as described by Toledo, F.C et al [17].

### Sperm morphology

To analyze the sperm morphology, a sperm smear was prepared on a glass slide from sperm solution, and the smears were air dried then stained by Gimsa, two hundred sperms were examined in each rat by light microscope (100 x, 400 x and 1000 x magnification). Sperms head, middle piece, tail defect and detached head were studied.

### Blood collection and serum sample

After anesthesia, blood was collected from the abdominal aorta; serum was separated by centrifugation (350 g, 20min, 4° C) and sera were stored at -80° C for subsequent assessment of hormone levels (Testosterone (T), Luteinizing Hormone (LH) and Follicle-Stimulating-Hormone (FSH)).

### Hormone assessment

Hormones level were measured by the ELISA using rat ELISA kits, with 450 nm wavelength and reference value 620 (Rat Testosterone ELISA kit, Cat No:CSB-E05100r, LH Cat No:CSB-E06869r, FSH Cat No:CSB-E12654r CUSABIO BIOTECH CO, LTD, Japan). The intra-assay variations were 5.2%, 6.8% and 6.3% for T, LH and FSH, and the kits sensitivity were 0.06 ng/ml, 0.15 mlu/ml and 0.4 mlu/ml respectively.

### Weight determination of the brain and reproductive organs

After blood collection, animals were sacrificed and their brain and reproductive organs (testes, seminal vesicle, vas deferens, ventral prostate glands and epididymis) were removed and their weight, absolute and relative were determined by digital scales with an accuracy 0.0001 g.

### Histomorphometric evaluation

Right testes of all animals (n=8 in each group) were taken and fixed in 4% paraformaldehyde. After fixation the samples were processed per standard protocols for paraffin embedding and were serially sectioned into 5µm sections. For each testis 5 cross-sections with at least 30µm distance from each other were selected, and stained with hematoxylin and eosin (H&E) [17], according to standard protocols. To analyze the average number of Sertoli cells per cross-section of tubules, according to Toledo F.C et al [17], the nuclei of Sertoli cells were counted in 20 seminiferous tubule round cross-sections per rat, with approximately equal diameter, at stage VII of spermatogenesis. In addition to obtain number of Spermatocytes and round Spermatids and their ratio to Sertoli cells, according to Dianne M. Creasy 1997 [18], one seminiferous tubule round cross-section per rat (with approximately equal diameter in all rats) were evaluated at stage VII of spermatogenesis. Finally to analyze diameter of the tubules, long and short diameter of 6 seminiferous tubules cross-sections per rat were measured at stage VII by ImageJ software (NIH) and mean of the diameter were reported. The data presented as median and interquartile intervals [Q1-Q3].

### Statistical analysis

Data analysis was performed using the SPSS 15.0 PC package (SPSS Inc., Chicago, IL). Data are expressed as the median and interquartile intervals [Q1-Q3]. Distributions between groups are compared using the Kruskal-Wallis test followed with Mann-Whitney test. Proportion of male offspring and mortality rate analyzed by Fisher exact test. A p < 0.05 was considered statistically significant.

## Results

### The percent of male offspring and mortality rate

The percentage of male offspring born from mothers treated with testosterone in both experimental groups І and П did not differ compared to their controls (Table S1). The mortality rate of the male offspring in both experimental groups did not differ significantly compared to their controls, at different days of examination (the most mortality in control rats was until day 30 of age) (Table S1).

### Anogenital distance, weight of the body, brain and reproductive organs

Anogenital distance (AGD) of the male offspring of both experimental groups did not differ significantly on different days of measurement, compared with male offspring of control groups (Table S2). The body weight of the male offspring in both experimental groups in comparison to control groups, at different ages is given in table S2.

The absolute and relative weights of the brain in the male offspring of experimental group І did not show significant differences in comparison to its controls however; in experimental group П, both weights were higher than that of control rats (Table S3).

Table S3 shows the absolute and relative weights of reproductive organs including testes, seminal vesicle, vas deferens, ventral prostate glands and epididymis in different groups.

### Sperm motility

Motility of the sperms in the male offspring of both experimental groups were significantly decreased in comparison to the control rats (p < 0.01). Type A and B motility of sperm in the male offspring of experimental group І were obviously decreased whereas, in the experimental group П type A and B showed no significant differences. Type C motility and D (Immotile sperm), in the male offspring of experimental group І increased compared to controls (p < 0.05 and p < 0.01, respectively) while in experimental group П, type C was not significantly different, but type D was increased (p < 0.01) (Figure S1).

### Sperm count and morphology

The number of sperm in male offspring of the experimental group І was significantly decreased, compared with control animals (p < 0.05), while in group П and its controls, animals showed no significant difference (Table S3). The sperm morphology was similar among all study groups, and no morphological abnormality was observed.

### Serum hormones levels

After puberty a significant reduction in serum concentration of testosterone was observed in male offspring of experimental group І compared with age-matched control animals (p < 0.05); there was no significant difference in LH and FSH concentrations between these two groups. No significant difference was found between male offspring of the experimental group П, in comparison to control one regarding hormonal levels (Figure S2).

### Histomorphometric parameters

The male offspring of the experimental group І expressed fewer Sertoli cell nuclei than control animals in the seminiferous tubules (p < 0.001), a difference however not observed in the experimental group П (Table S3 and Figure S3). Cell counts of Spermatocyte and round Spermatid in the offspring of experimental group І were significantly decreased and seminiferous tubules diameter was not significantly different in each experimental group compared to its controls (table S3).

## Discussion

The present study demonstrated that prenatal exposure of male rat fetuses to excess testosterone disrupted reproductive function, an effect highly dependent on the time, duration and level of exposure. We found that the exposure to the exogenous testosterone, during embryonic days 16-19, is associated with disruption of the reproductive system of male rats in terms of reduction of the testis’s weight, number of Sertoli cells, Spermatocyte and Spermatid cells, sperm count and motility as well as declining of the serum concentration of testosterone after puberty. Except for reduction of sperm motility, the other effects were not observed in male rats exposed to exogenous testosterone on day 20 of their fetal life.

Previous studies have showed that exposing the female animals to exogenous androgen during fetal life can cause changes in female reproductive phenotype in adulthood. It was further shown that the symptoms of polycystic ovary syndrome appear after puberty in animals (monkeys, sheep and rats) exposed to androgen, prenatally or postnatally [3]. Although several studies demonstrate the impact of prenatal exposure of female fetuses to excess testosterone, data on male offspring are limited.

Proliferation of Sertoli cells in rats start from fetal life and continues until weeks 2-3 after birth [17] and male fetuses of rats produce an androgen surge, beginning on embryonic day 16 and lasting until embryonic day 21, its peak being around the critical developmental window (approximately postnatal days 18-19) [19]. In the current study in the offspring of animals exposed to exogenous testosterone on days 16-19 fetal life, the number of Sertoli cells in testis were reduced after puberty. In our research, based on similar human studies [12], we assumed that injection of exogenous testosterone to pregnant animals, intra testicular testosterone level in male fetuses during their testes development (proliferation of Sertoli cells), is reduced and consequently, the reduction of the number of Sertoli cells occurred, because androgens play a great role in proliferation of Sertoli cells, expression of some of the genes of Sertoli cells and supporting meiotic and post-meiotic development of germinal cells [20]. It has been shown that the number of Sertoli cells decreased in mice without androgen receptors [21] or rats not exposed to androgen during the period of testicular development in fetal life [13]. In another study carried out on rams reported different result; prenatal exposure of male fetuses to exogenous androgen increased the number of Sertoli cells [11]. These different observations may be explained by difference of the strain, time, duration, level of prenatal exposure and type of androgen.

Hormones play a vital role in development and preservation of male reproductive function. In the present study, a reduction of serum concentration of testosterone was observed in adult offspring of experimental group І, while their LH and FSH serum levels were similar to their controls. Reduction of testosterone level is probably due to the reduction of number of Sertoli cells, since the function and survival of Leydig cells in adulthood is dependent on persistent presence of Sertoli cells [22,23]. Furthermore it has been shown that prenatal exposure of male rat to Di-n-Butyl Phthalate (DBP) (anti-androgen, which reduces androgen level) decreases the activity level of testicular steroidogenic enzymes, as well as testosterone level in adults [16]. Another study demonstrated that prenatal exposure of male rat to testosterone propionate, during days 17-19 of gestation, will lead to reduction of plasma testosterone level during adulthood [10]. Rams exposed to testosterone during their development, showed a higher level of plasma testosterone at 20 weeks of age, while a lower level had been seen at the age of 30 weeks [24]. It seems that prenatal excess androgen may lead to reduction of testosterone level after puberty. However in human studies, the serum concentration of testosterone in boys born to mothers with polycystic ovary syndrome was not significantly different from the offspring of healthy women [25], results not in agreement with ours; this difference could be explained by the fact that these boys were exposed to androgen excess throughout their fetal life, whereas in our study fetuses were exposed to exogenous androgen only during a specific period of their fetal life.

In our research, despite the reduction of Sertoli cells number in the offspring of experimental group І, serum FSH concentration showed no difference, compared to controls, which may be due to a possible decline in FSH receptors expression and Sertoli cells sensitivity to FSH, due to reduction of testosterone level. This has been reported in adult mice lacking androgen receptors, there was a reduction in FSH receptor expression, indicating that androgens can play a role in regulating FSH receptor in adult animal; therefore, it is possible that one of the trophic effects of androgen on the Sertoli cell is to increase sensitivity to FSH [26].

The current study shows a decrease on the sperm count as well as reduction of the number of Spermatocyte and round Spermatid cells in the offspring of experimental group І, which may be explained by reduction of the Sertoli cells or testosterone levels, as both have a vital role in spermatogenesis. It has been shown that Sertoli cells play a crucial role in proliferation and development of primordial germ cells during fetal life, preserving the process of spermatogenesis [27,28] and providing a suitable environment for proliferation and maturation of germinal cells [17]. Testosterone is also known as a prohibiting factor for death of germinal cells [11]. Occlusion of seminiferous tubules due to increase of androgen in prenatal period has been also suggested by others as a possible mechanism of sperm count reduction [1]; however this has not been examined in the present study. One study of sheep supported the negative effect of prenatal exposure to androgen excess on the quality and quantity of sperm after puberty [1]. Significant reduction of sperm count and fertility was observed in men with congenital adrenal hyperplasia, exposed to high amounts of androgens during their fetal life [29,30]; however it was not reported in boys born from mothers with polycystic ovary syndrome (PCOS) [25]. Although men exposed to high androgens during the fetal life, have different presentations after puberty, possibly explained by different types of androgens resulting from the mentioned early [25,29,30], another reason is because the placenta converts the androgen to estrogen via aromatase enzyme [1] probably, in sons of women with polycystic ovary syndrome the amount of androgen metabolized by placenta, while the male fetus with congenital adrenal hyperplasia, fetus is exposed to androgen, directly, during fetal life.

In the present study, in offspring of both experimental groups exposed to testosterone during their fetal life, reduction of sperm motility was observed, perhaps due to the disruption in development of epididymis by fetal exposure to the androgen; however this needs more investigation. In another research conducted on male sheep, it was concluded that exposing the animal to exogenous androgen in a particular period of fetal development, leads to a reduction of sperm motility at the time of puberty [1]. A similar study, also conducted on rams exposed to dihydrotestosterone during prenatal life, showed significant decline in motility of sperm; however in those who were exposed to testosterone, no changes were noticed [9].

In this study, we observed brain weight in the offspring of the experimental group ІІ was higher than its controls; it seems that testosterone has stronger effect on brain size and structure during perinatal life. At birth, the nervous system of rats are in the medium-term of differentiation and development and are sensitive to androgen. The influence of fetal levels of testosterone was tested indirectly by comparing brain size of 9-year old dizygotic twins, irrespective of their own sex and children with a male vs. female co-twin had slightly larger brains [31].

In the current study, decrease in the absolute weight of testes, possibly due to the reduction of the testosterone level, was observed in the offspring of group І; body weight of the male offspring in the both experimental groups showed no significant difference compared with their controls. On the other hand, in another study of sheep, in male animals exposed to androgen during prenatal life, body weight was significantly decreased compared with controls [1]. In another research the body weight of sons of mothers with polycystic ovary syndrome was higher than those normal mothers [25]. We found no convincing reason for this difference and further investigation is needed.

In this study, except for reduction of sperm motility, the other effects (reduction of Sertoli cells, Spermatocyte and Spermatid, sperm count and testosterone concentration), were not observed in male rats exposed to exogenous testosterone at day 20 of their fetal life; this could be explained by the difference in time exposure to androgen as in experimental group І it was administered during the male programming window [14], and time of first peak of sexual differentiation of hypothalamus [10], therefore resulted in more severe effects on reproductive function. The rats of experimental group П were exposed to exogenous testosterone on the last day of the gestational period, therefore the hypothalamus-pituitary-gonad axis had been programmed before.

The main strengths of the present study, is the examination of the effect of prenatal exposure of two different doses of testosterone, at different gestational ages, on the male reproductive system. One limitation is that we did not measure of intra testicular and serum levels of testosterone in fetal life; or at the time birth. Another limitation is, both prenatal exposure time and doses were changed simultaneously, as a result of which it is not possible to separate the influence of different doses or exposure times.

## Conclusion

Our study demonstrated that the reproductive system is susceptible to androgen excess insults during critical developmental periods. Therefore it seems that the reproductive system in individuals exposed to high levels of androgens during fetal life should be evaluated at puberty, and likely to be treated.

## Supporting Information

Figure S1 **(A-E) Sperm motility assessment of study groups at 120-130 days of age (n=8 per group).** A; comparison of the motile sperm percent between experimental group and its control, B; comparison of the percent of type A sperm (motile with progressive trajectory) between experimental group and its control, C; comparison of the percent of type B sperm (motile with non-progressive trajectory) between experimental group and its control, D; comparison of the percent of type C sperm (sluggish) between experimental group and its control, E; comparison of the percent of type D sperm (immotile) between experimental group and its control. Values are expressed as median and interquartile intervals, Mann-Whitney test, * p < 0.05, ** p < 0.01.
(TIF)

Figure S2 **(A–D) The hormonal profiles of study groups at 120-130 days of age (n=8 per group).** A; comparison of testosterone concentration between experimental group and its control, B; comparison of luteinizing hormone concentration between experimental group and its control, C; comparison of follicle-stimulating-hormone concentration between experimental group and its control, D; comparison of the ratio of concentration luteinizing hormone to follicle-stimulating-hormone between experimental group and its control. Values are expressed as median and interquartile intervals Q1 – Q3. Mann-Whitney test, * p < 0.05.
(TIF)

Figure S3 **(A–H) Histopathological analysis of testis in study groups at 120-130 days of age (n=8 per group).** H&E stain. A and B; seminiferous tubules cross-sections in control group I (40 x, 400 x magnification, respectively), C and D; seminiferous tubules cross-sections in experimental group I (40 x, 400 x magnification, respectively), E and F; seminiferous tubules cross-sections in control group П (40 x, 400 x magnification, respectively), G and H; seminiferous tubules in experimental group П (40 x, 400 x magnification, respectively). The number of Sertoli cells in the experimental group compared to its controls. ↑ arrow = Sertoli cell nucleous, ↑ black arrow = Spermatocyte cell nucleous and white arrow = round Spermatid nucleous.
(TIF)

Table S1 **Proportion of male offspring in study groups at the first day of birth and mortality rate until days 30, 60 and 120 of age.** Fisher exact test.
(DOCX)

Table S2 **Anogenital distance (AGD) and body weight of male offspring on different days.** Values are expressed as median and interquartile intervals [Q1 –Q3]. Mann - Whitney Test. * p < 0.05, ** p < 0.01.
(DOCX)

Table S3 **The absolute and relative weights of brain and reproductive organs, Sertoli, Spermatocyte, Spermatid cell number, tubules diameter and sperm count of study groups between 120–130 days of age.** Values are expressed as median and interquartile intervals [Q1 –Q3]. Mann-Whitney Test. * P < 0.05, ** p < 0.01, *** p < 0.001. Relative weight is organ weight/body weight (g).
(DOCX)
