# Supplementary figures and images for: Prenatal Testosterone Exposure Worsen the Reproductive Performance of Male Rat at Adulthood
Source: PLoS One. 2013 Aug 15;8(8):e71705. doi: 10.1371/journal.pone.0071705 (PMC3744450; doi:10.1371/journal.pone.0071705)

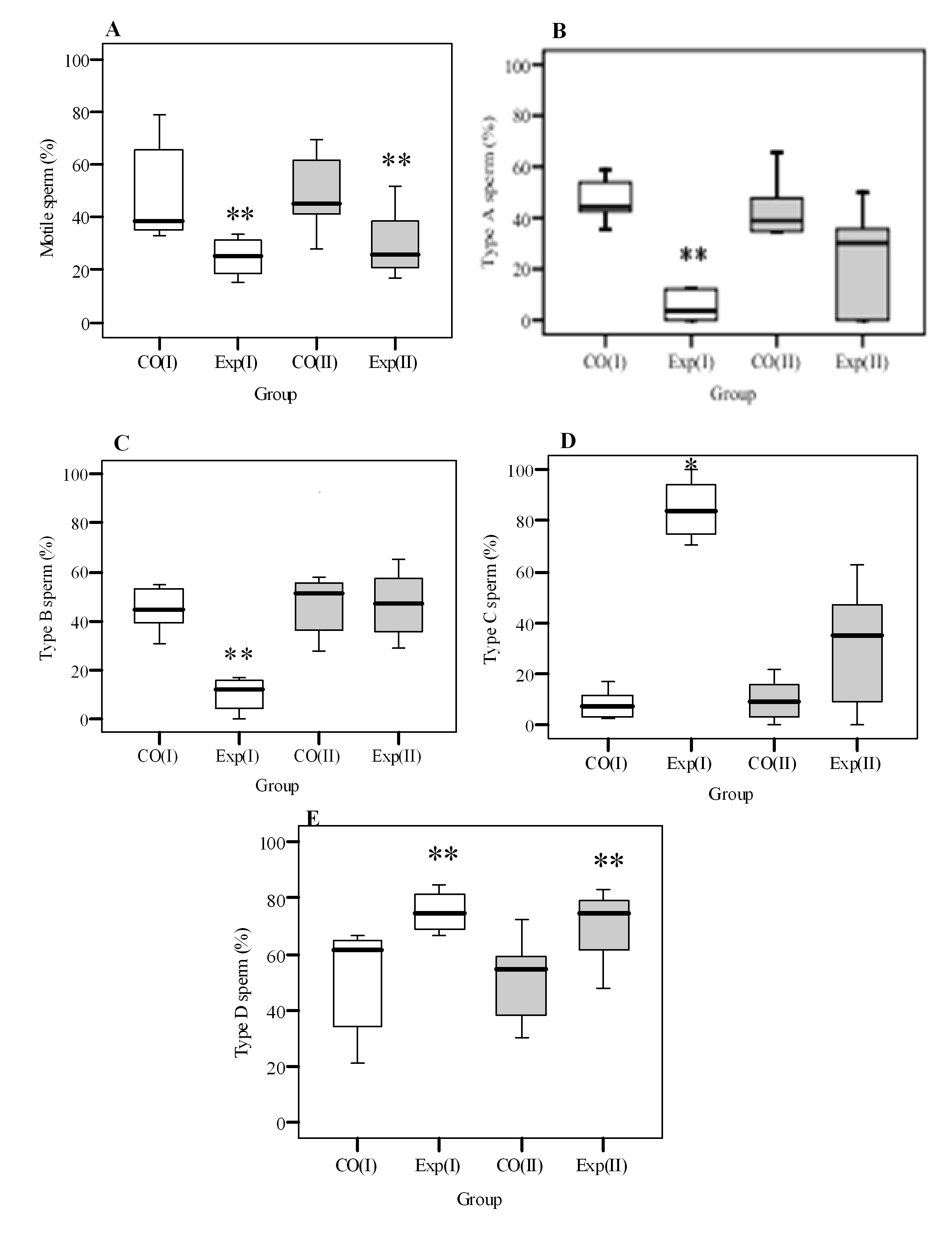

Supplement: Figure S1 — (A-E) Sperm motility assessment of study groups at 120-130 days of age (n=8 per group). A; comparison of the motile sperm percent between experimental group and its control, B; comparison of the percent of type A sperm (motile with progressive trajectory) between experimental group and its control, C; comparison of the percent of type B sperm (motile with non-progressive trajectory) between experimental group and its control, D; comparison of the percent of type C sperm (sluggish) between experimental group and its control, E; comparison of the percent of type D sperm (immotile) between experimental group and its control. Values are expressed as median and interquartile intervals, Mann-Whitney test, * p < 0.05, ** p < 0.01. (TIF) [file pone.0071705.s001.tif]

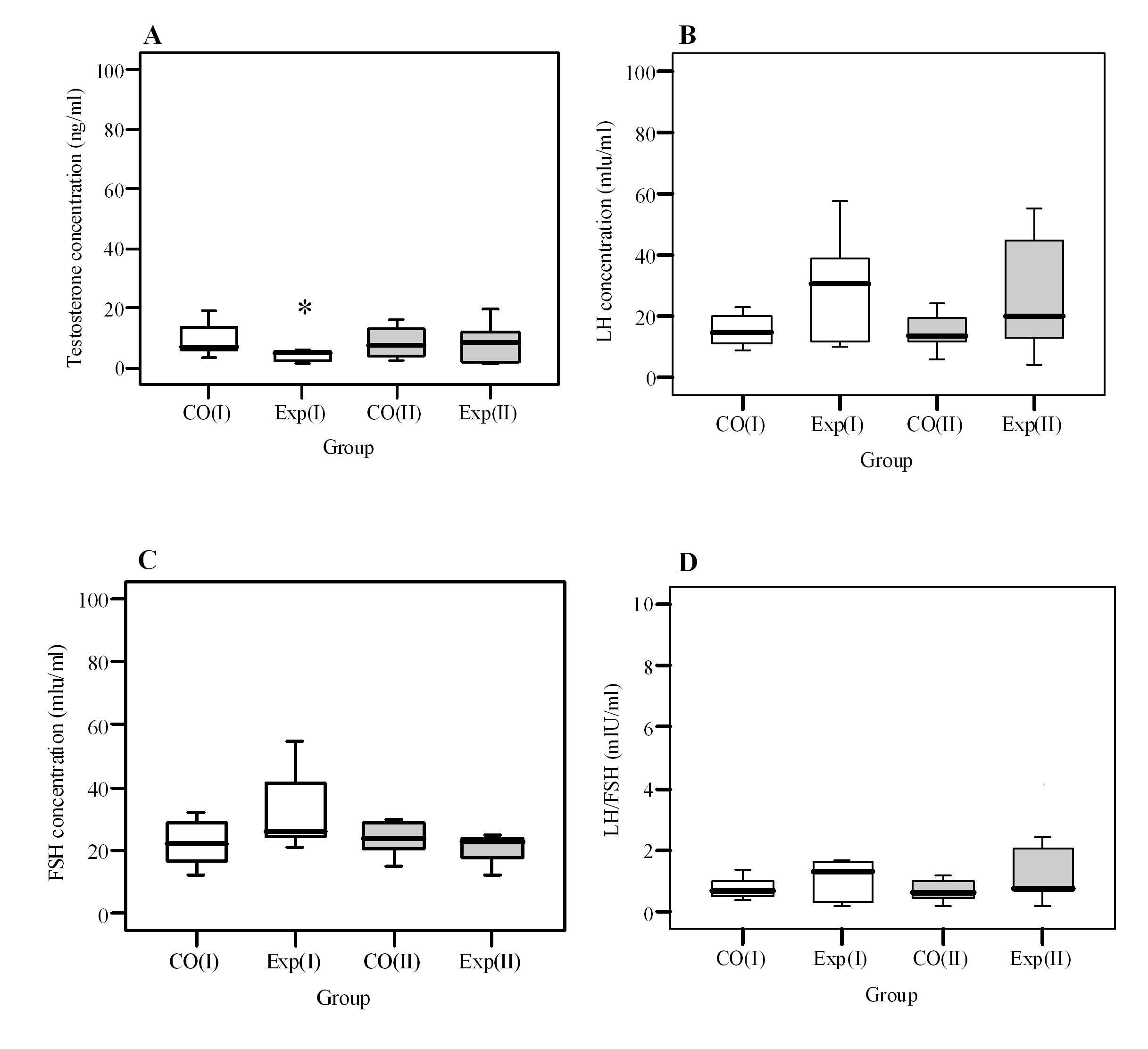

Supplement: Figure S2 — (A–D) The hormonal profiles of study groups at 120-130 days of age (n=8 per group). A; comparison of testosterone concentration between experimental group and its control, B; comparison of luteinizing hormone concentration between experimental group and its control, C; comparison of follicle-stimulating-hormone concentration between experimental group and its control, D; comparison of the ratio of concentration luteinizing hormone to follicle-stimulating-hormone between experimental group and its control. Values are expressed as median and interquartile intervals Q1 – Q3. Mann-Whitney test, * p < 0.05. (TIF) [file pone.0071705.s002.tif]

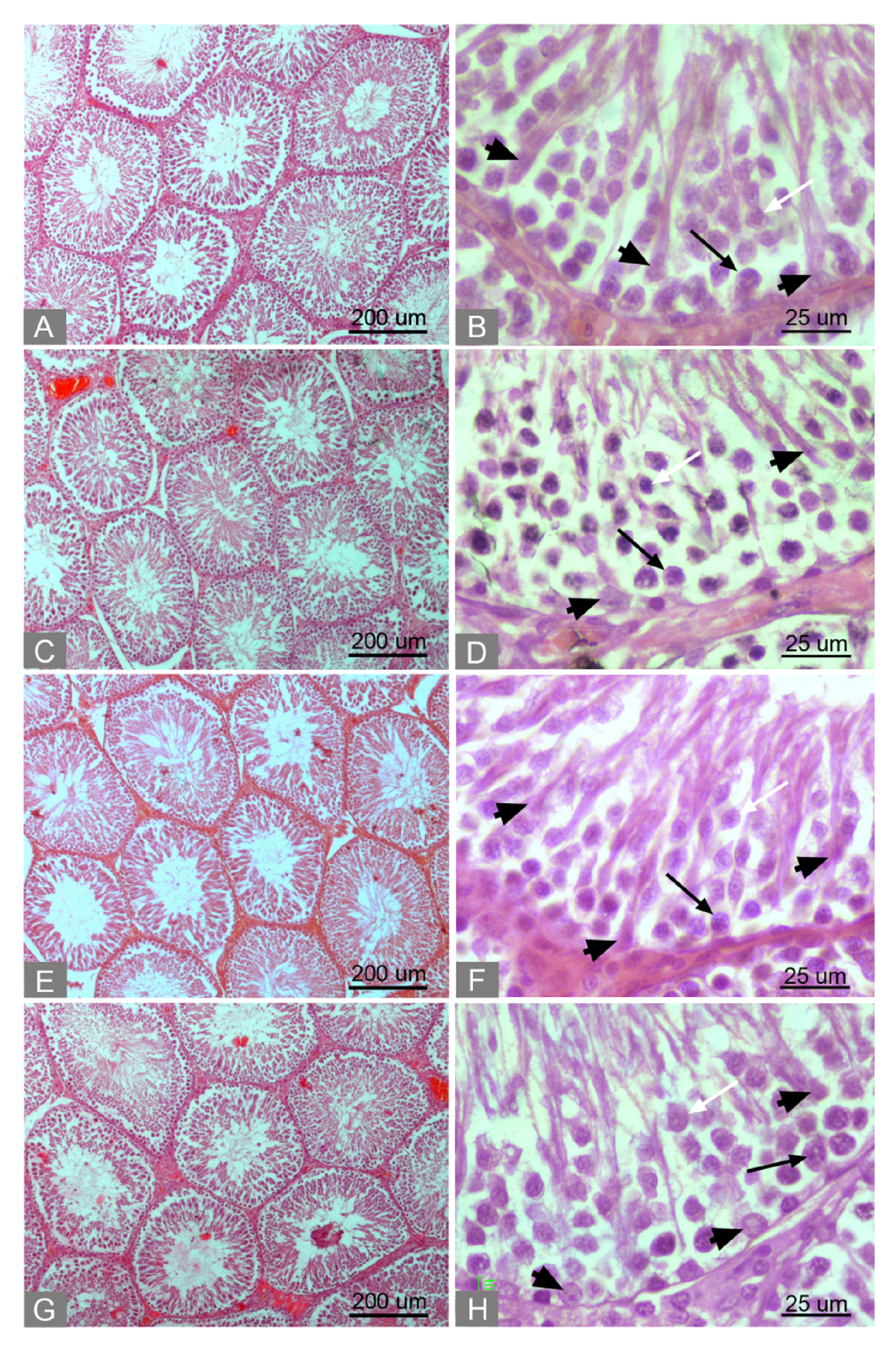

Supplement: Figure S3 — (A–H) Histopathological analysis of testis in study groups at 120-130 days of age (n=8 per group). H&E stain. A and B; seminiferous tubules cross-sections in control group I (40 x, 400 x magnification, respectively), C and D; seminiferous tubules cross-sections in experimental group I (40 x, 400 x magnification, respectively), E and F; seminiferous tubules cross-sections in control group П (40 x, 400 x magnification, respectively), G and H; seminiferous tubules in experimental group П (40 x, 400 x magnification, respectively). The number of Sertoli cells in the experimental group compared to its controls. ↑ arrow = Sertoli cell nucleous, ↑ black arrow = Spermatocyte cell nucleous and white arrow = round Spermatid nucleous. (TIF) [file pone.0071705.s003.tif]
