# Supplementary material for: Prenatal Testosterone Exposure Worsen the Reproductive Performance of Male Rat at Adulthood
Source: PLoS One. 2013 Aug 15;8(8):e71705. doi: 10.1371/journal.pone.0071705 (PMC3744450; doi:10.1371/journal.pone.0071705)
Supplement: Table S1 — Proportion of male offspring in study groups at the first day of birth and mortality rate until days 30, 60 and 120 of age. Fisher exact test. (DOCX) [file pone.0071705.s004.docx]

| Parameters | Groups | | | |
| --- | --- | --- | --- | --- |
|  | Control (I)  N (%) | Experimental (I)  N (%) | Control (Π)  N (%) | Experimental (Π)  N (%) |
| Number of male offspring/total offspring at the first day of birth | 19/42 (45.2) | 22/49 (44.9) | 23/39 (59.0) | 12/30 (40) |
| Mortality until day 30 | 3/19 (15.8) | 7/22 (31.8) | 6/23 (26.1) | 0 |
| Mortality until day 60 | 5/19 (26.3) | 11/22 (50.0) | 6/23 (26.1) | 0 |
| Mortality until day 120 | 6/19 (31.6) | 12/22 (54.5) | 6/23 (26.1) | 0 |
