# Supplementary material for: Prenatal Testosterone Exposure Worsen the Reproductive Performance of Male Rat at Adulthood
Source: PLoS One. 2013 Aug 15;8(8):e71705. doi: 10.1371/journal.pone.0071705 (PMC3744450; doi:10.1371/journal.pone.0071705)
Supplement: Table S2 — Anogenital distance (AGD) and body weight of male offspring on different days. Values are expressed as median and interquartile intervals [Q1 –Q3]. Mann - Whitney Test. * p < 0.05, ** p < 0.01. (DOCX) [file pone.0071705.s005.docx]

| Parameters | Groups | | | |
| --- | --- | --- | --- | --- |
|  | Control (I)  n=8 | Experimental (I)  n=8 | Control (Π)  n=8 | Experimental (Π)  n=8 |
| AGD on day 6 (mm) | 5.0 [4.0 -5.7] | 5.0 [5.0 -6.0] | 5.0 [5.0 -5.7] | 5.0 [5.0 -6.0] |
| AGD on day 30 (mm) | 14.0 [12.5 -15] | 15.0 [14.2 -16.0] | 15.0 [14.0 -18.7] | 15.0 [14.2 -16.0] |
| AGD on day 60 (mm) | 31.0 [28.2 -34.2] | 32.0 [30.0 -34.0] | 30.5 [29.0 -33.5] | 30.0 [30.0 -32.7] |
| AGD after puberty (mm) | 33.5 [30.5 -35.7] | 34.5 [34.0 -35.7] | 33.5 [33.0- 36.05] | 34.0 [32.2 -35.0] |
| Body weight on birth day (g) | 6.1 [5.5 -6.3] | 6.6 [6.3 -7.0] ** | 6.3 [5.9 -7.4] | 6.2 [5.6 -6.4] |
| Body weight on day 15 (g) | 18.8 [18.1 -19.9] | 19.2 [17.8 -21.6] | 23.2 [22.0 -25.3] | 27.0 [24.3 -31.5] * |
| Body weight on day 30 (g) | 49 [43.7 -52.7] | 50.5 [48.2 -53.2] | 68.5 [60.75 -77.0] | 60.0 [57.2 -62.0] * |
| Body weight on day 45 (g) | 105 [99.2 -114.2] | 89.0 [84.0 -95.0] * | 110.0 [107.5 -118.7] | 99.00 [84.7 -109.5] * |
| Body weight on day 60 (g) | 162.5 [138.5 -180.2] | 137.5 [118.2 -151.2] * | 169.5 [152.2 -214.5] | 150.5 [139.7 -157.7] * |
| Body weight after puberty | 312.0 [292.2- 345.7] | 332.0 [302.2- 351.5] | 321.0 [311.0 -346.0] | 338.0 [323.7 -381.2] |
