# Supplementary material for: Prenatal Testosterone Exposure Worsen the Reproductive Performance of Male Rat at Adulthood
Source: PLoS One. 2013 Aug 15;8(8):e71705. doi: 10.1371/journal.pone.0071705 (PMC3744450; doi:10.1371/journal.pone.0071705)
Supplement: Table S3 — The absolute and relative weights of brain and reproductive organs, Sertoli, Spermatocyte, Spermatid cell number, tubules diameter and sperm count of study groups between 120–130 days of age. Values are expressed as median and interquartile intervals [Q1 –Q3]. Mann-Whitney Test. * P < 0.05, ** p < 0.01, *** p < 0.001. Relative weight is organ weight/body weight (g). (DOCX) [file pone.0071705.s006.docx]

| Parameters | Control (I)  n=8 | Groups  Experimental (I)  n=8 | Control (Π)  n=8 | Experimental (Π)  n=8 |
| --- | --- | --- | --- | --- |
| Brain (g) | 1.7[1.7 -1.8] | 1.8[1.8 -1.9] | 1.7[1.6 -1.7] | 1.9[1.9 -2.1] ** |
| Relative to body weight (g) | 0.005[0.005 -0.006] | 0.005 [0.005-0.006] | 0.005[0.005 -0.005] | 0.005[0.005 -0.006] * |
| Testes (pair) (g) | 3.1 [3.0 -3.4] | 2.9[2.8 -3.1] * | 2.8 [2.4 -3.4] | 3.4 [3.3 - 3.5] |
| Relative to body weight (g) | 0.009[0.009 -0.01] | 0.009[0.008 -0.009] | 0.009[0.008 -0.01] | 0.01[0.009 -0.01] |
| Seminal vesicle (g) | 1.7 [1.6 -2.4] | 2.2 [1.7 -2.2] | 1.7 [1.5 -1.9] | 1.7 [1.6 -2.1] |
| Relative to body weight (g) | 0.005[0.005 -0.006] | 0.006[0.005 -0.007] | 0.005[0.004 -0.006] | 0.005[0.004 -0.006] |
| Vas deferens (pair) (g) | 0.2[0.2 -0.2] | 0.2[0.2 -0.2] | 0.2[0.2 -0.2] | 0.2[0.2 -0.2] |
| Relative to body weight (g) | 0.0008[0.0008 -0.001] | 0.0008[0.0007 -0.0009] | 0.0008[0.0008 -0.0009] | 0.0008[0.0007 -0.0008] |
| Prostate glands(g) | 0.5[0.5 -0.6] | 0.6[0.5 -0.6] | 0.5[0.4 -0.6] | 0.6[0.5 -0.6] |
| Relative to body weight (g) | 0.001 [0.001 -0.002] | 0.001[0.001 -0.002] | 0.001[0.001 -0.002] | 0.001[0.001 -0.001] |
| Epididymis (pair) (g) | 2.3 [1.7 - 3.2] | 1.6[1.4 - 2.0] | 1.8 [1.3 -3.2] | 1.8 [1.5 - 3.0] |
| Relative to body weight (g) | 0.007[0.005 -0.009] | 0.004[0.004 -0.005] * | 0.005[0.004 -0.009] | 0.005[0.004 -0.008] |
| Sertoli cell number | 12.2[11.6 -12.5] | 5.4[4.5 -5.6] *** | 12.6[11.9 -13.4] | 12.6[11.7 -13.4] |
| Sertoli cell number/Spermatocyte cell number | 0.13[0.11 -0.17] | 0.09[0.08 -0.11] * | 0.13[0.11 -0.16] | 0.15[0.11 -0.17] |
| Sertoli cell number/Spermatid cell number | 0.06[0.04 -0.07] | 0.04[0.03 -0.04] * | 0.05[0.04 -0.07] | 0.05[0.04 -0.08] |
| Tubules diameter (µm) | 336.5[300.2 -353.5] | 327.8[320.4 -343.0] | 336.0[329.2 -344.8] | 323.1[300.7 -338.0] |
| Sperm count (×10^6^) / ml | 100.5[63.2 -140.2] | 56.0[35.7 -71.3] * | 94.5[78.1 -222.7] | 110.2[73.3 -129.2] |
